# Predictors of Breast Cancer Screening Behaviors Among American Indian Women in the Northern Plains

**DOI:** 10.1093/geroni/igaa057.354

**Published:** 2020-12-16

**Authors:** Soonhee Roh, Yeon-Shim Lee, Heehyul Moon

**Affiliations:** 1 University of South Dakota, Sioux Falls, South Dakota, United States; 2 San Francisco State University, San Francisco, California, United States; 3 University of Louisville, Louisville, Kentucky, United States

## Abstract

Purpose: This study examined predictive models of the utilization of mammograms among American Indian women adapting Andersen’s behavioral model. Using a sample of 143 American Indian women residing in the Northern Plains. Methods: Data were collected using a self-administered survey completed by 143American Indian women over the age of 45 in the Midwest. Logistic regression analyses were conducted to assess predisposing (age and marital status), need (personal and family cancer history), and enabling factors (education, monthly household income, mammogram screening awareness, breast cancer knowledge, self-rated health, and cultural practice to breast cancer screening). Results: Nested logistic regression analyses indicated that only 55.5% of participants reported having had a breast cancer screening within the past 2 years, whereas 21.0% never had a mammogram test. After controlling for predisposing and need factors, higher education, greater awareness of mammogram, and higher utilization of traditional Native American approaches were significant predictors of mammogram uptake. Conclusions: The findings highlight important implications for intervention strategies aimed at improving breast cancer screening and service use among American Indian women. Educating health professionals and American Indian community members about the importance of breast cancer screening is highly needed. It is critical to assess a woman’s level of traditional beliefs and practices and its possible influence on screening participation and future screening intention. Given the findings, prevention and intervention strategies, including public awareness and education about breast cancer screening are promising avenues to reduce screening disparities among American Indian women.

